# An Accelerated Editing Method for Stress Signal on Combine Harvester Chassis Using Wavelet Transform

**DOI:** 10.3390/s25134100

**Published:** 2025-06-30

**Authors:** Shengcao Huang, Zihan Yang, Zhenghe Song, Zhiwei Yu, Xiaobo Guo, Du Chen

**Affiliations:** 1 College of Engineering, China Agricultural University, Beijing 100083, China; huangshengcao@chiaic.com (S.H.); songzhenghe@cau.edu.cn (Z.S.); 2 Luoyang Smart Agricultural Equipment Institute Co., Ltd., Luoyang 471000, China; yuzhiwei@chiaic.com; 3 State Key Laboratory of Intelligent Agricultural Power Equipment, Luoyang 471000, China

**Keywords:** combine harvester, load spectrum, acceleration editing, wavelet transform

## Abstract

This paper presents a load spectrum acceleration editing method based on wavelet transform. The principle of the method is to decompose the target signal using wavelet transform to obtain high-frequency wavelet components, which are classified and combined based on their frequency components for accelerated editing. During the damage segment identification stage, a threshold selection method based on the pseudo-damage gradient of the segment identification results is proposed. An envelope-based damage identification method is used to extract high-damage segments from the original signal, which are then concatenated to form an accelerated signal. Using the stress signal on the chassis of a combine harvester as a case study, the effectiveness of various accelerated editing methods is compared, with a discussion on the selection of wavelet function parameters. The results indicate that, compared to the time-domain damage retention method and the traditional wavelet transform accelerated editing method, the proposed improvement enhances the acceleration effect of the time-domain signal by 7.76% and 15.92%, respectively. The accelerated signal is consistent with the original signal in terms of statistical parameters and power spectral density. Additionally, we also found that an appropriate selection of the wavelet function’s vanishing moment can further reduce the time-domain signal length of the accelerated result by 4.8%. This study can provide beneficial experiential references for load spectrum development in the accelerated durability testing of agricultural machinery.

## 1. Introduction

Improving quality and efficiency has always been the optimization goal of agricultural machinery enterprises for their products [1]. As a representative of complex agricultural machinery, the combine harvester not only fulfills its transport function but also needs to allocate power efficiently to various crop processing units, such as the feeding device, threshing and separating device, and cleaning device, to drive the interactions between operational components and crops [2]. During the field operation of combine harvesters, component structural failures and transmission system faults often directly affect the machine’s operational performance and harvesting efficiency. Research indicates that delayed harvesting not only affects the current season’s yield but can also have irreversible effects on the next phase of agricultural production [3]. Therefore, the durability and reliability indicators of combine harvesters are a common focus for both agricultural machinery companies and agricultural producers.

Since 1932, when Kloth and Stroppel successfully obtained the load spectrum for tractor power take-off shafts [4], research on load spectrum has long supported the design and validation of agricultural machinery, laying the foundation for producing efficient and reliable equipment and driving the development of new technologies in the field, such as power shift and CVT technology [5]. Today, with the increasing competition in the global market, agricultural machinery companies must obtain load spectra that are closer to the actual working conditions and operational requirements of users, thereby ensuring a more precise assessment of whether the machinery products satisfy the design goals for durability and reliability [6]. At the same time, the ever-compressed product development cycle further requires higher demands from the product test engineers. The good news is that with the continuous development of electro-hydraulic servo systems, more convenient technical means are now available for conducting durability tests on agricultural machinery in laboratory environments [7,8]. However, fully reproducing the load spectrum obtained from field tests on a test bench, although it can accurately reflect the actual loaded condition, requires a high investment of time and economic cost. To ensure consistency in the loading effects of load spectrum between field tests and indoor bench tests, while further improving testing efficiency and reducing costs, it is necessary to study accelerated load spectrum editing methods based on the measured load characteristics [9]. Many scholars have researched accelerated load spectrum editing methods from the perspectives of the time domain and frequency domain. Dividing the signal into time windows of specific lengths and setting thresholds based on the damage proportion caused by loads within each window is a classic approach widely used in time-domain acceleration methods. This method has been successfully applied in commercial software [10]. Based on the above method framework, Conle et al. utilized a stress–strain amplitude editing method to remove load segments with minor fatigue damage, achieving accelerated editing of load time-domain signals [11]. Lin et al. analyzed the loads on motorcycle components using a time-domain damage retention method, establishing a complete durability analysis case from load spectrum acquisition to bench validation [12]. In the vehicle field, Yu et al. conducted applied research on durability testing of complete machines and components using time-domain accelerated editing methods [13,14,15]. Paraforos et al. applied this editing method to the field of agricultural machinery, combining the material S-N curve of the tested component to identify the time-domain damage segments, and completed accelerated testing of a four-rotor swather [16]. Chen et al. performed accelerated editing of a subway mounting frame based on the multi-channel damage correlation editing theory and validated the feasibility of this method in fatigue life prediction [17].

The accelerated editing method based on time-domain damage retention is simple to calculate and highly applicable. However, the fixed window length limits this method’s ability to balance the precision of damage segment identification and the effectiveness of time-domain reduction. The frequency-domain accelerated editing method transforms time-domain signals into the time-frequency domain and performs statistical analysis of frequency-domain amplitudes within a given time window to identify time intervals below a set threshold [18,19]. Abdullah et al. [20] used the power spectrum distribution obtained by short-time Fourier transform as a basis to identify and remove small loads in the load spectrum, resulting in an accelerated signal that almost completely retained the original damage. Zhu et al. [21] applied short-time Fourier transform to accelerate the editing of automotive component loads, demonstrating that the accelerated signal obtained through this method is shorter in duration and statistically closer to the original load signal compared to time-domain editing methods. The frequency-domain accelerated editing method is not constrained by fixed window intervals, allowing for the more precise time-domain identification of retained segments. However, its effectiveness strictly depends on the correlation between frequency-domain energy amplitudes and fatigue damage.

Zheng et al. [22] proposed a load spectrum acceleration editing method based on wavelet transform, which can be considered a combination of time-domain and frequency-domain acceleration techniques. This method employs wavelet decomposition to extract components at different frequencies, simultaneously establishing a connection between the component features and the time-domain damage intervals. The effectiveness of the method was demonstrated through a comparative validation using the suspension load of an automotive powertrain. Li et al. [23] focused on the load of the tractor power take-off as the research object and conducted applied research based on the aforementioned method. Liu et al. [24] used a time-frequency editing method based on wavelet transform to accelerate the editing of measured load spectrum for rubber isolators. The results show that the accelerated spectrum after editing produces the same damage distribution calculation results for the tested sample as the original load spectrum. The operating environment of the combine harvester in the field is harsh, the loading conditions of the working components are complex, and there are load components with significant differences in time-frequency characteristics. Therefore, by decomposing the load signal using wavelet transform and identifying and filtering out the low-damage segments of each component, this technical approach may have potential advantages in the accelerated editing of load for combine harvester working components.

In summary, this paper takes the stress signal of the combine harvester chassis as an example to study the load spectrum acceleration editing method based on wavelet transform. The wavelet components involved in the accelerated editing of traditional methods are classified and constrained, and on this basis, the threshold division method based on maximum amplitude is optimized. By constructing an association model between the threshold of each component and the damage identification results, the threshold levels are set using pseudo-damage gradients. Furthermore, this paper discusses the impact of key parameter selection on the results of accelerated editing.

The remaining structure of this paper is arranged as follows: Section 2 presents the testing system and field experiments for obtaining the stress of the combine harvester chassis, and it analyzes the signal data in conjunction with the operational characteristics of the harvester. Section 3 explains the theoretical background of the load spectrum acceleration editing method based on wavelet transform and discusses the results obtained using conventional methods. Section 4 introduces the optimization ideas of this paper for the traditional wavelet transform-based accelerated editing method, specifically including the classification of wavelet components and optimization of the threshold selection method. Finally, in Section 5, the results of accelerated editing obtained by different methods are compared to verify the superiority of the proposed method, the impact of the choice of wavelet function vanishing moments in the improved method on acceleration results is discussed, and application recommendations are provided.

## 2. Materials and Methods

### 2.1. Measurement System

**Figure 1:**
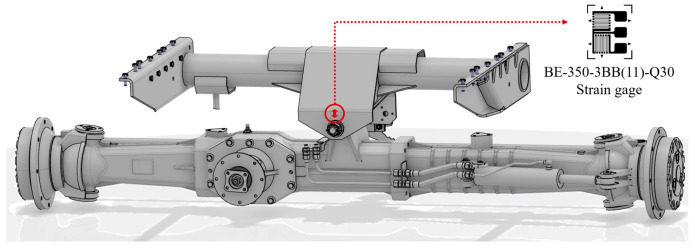
Schematic diagram of strain gauge location.

The experimental prototype in this study is a large-scale dual longitudinal axial-flow combine harvester. The measurement point is selected at the hinge point between the chassis rear axle and the body to monitor the variation in the supporting force exerted by the rear axle on various operational components of the harvester. The testing system consists of strain gauges and a data acquisition device. The relevant hardware parameters of the testing system are shown in Table 1. Figure 1 shows the structure of the area at the measurement point and a schematic diagram of strain gauge installation.

### 2.2. Signal Characteristic Analysis

**Figure 2:**
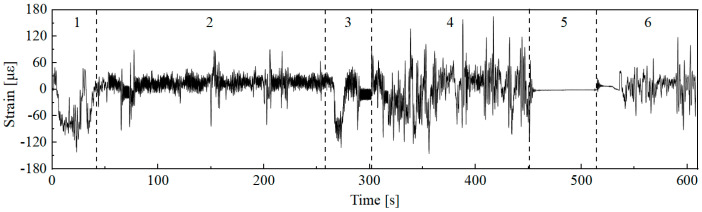
Measured signal for field operations.

The time-domain signal after preprocessing operations such as de-burring and drift removal is shown in Figure 2. The total length of the data sample is 608 s, with a sampling frequency of 1024 Hz. Based on the actual field operations of the combine harvester, the working conditions are divided into startup adjustment, normal harvesting, field reversing, field driving with a full tank, grain unloading, and field driving with an empty tank after unloading, corresponding to signal segments 1 to 6 in Figure 2. By analyzing the driving video recorded in the harvester cab during field testing, the time points of each operating condition were accurately identified. In addition, no harvester blockages or unexpected breakdowns occurred in the actual operating scenarios corresponding to the sample data used in this study.

**Figure 3:**
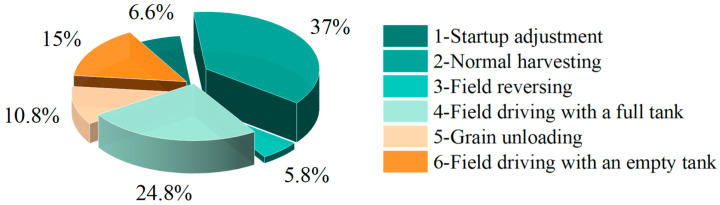
Time distribution of combine harvester conditions.

Initially, the full dataset is segmented into different operating condition datasets based on the division shown in Figure 2. The time proportion for each condition is then calculated by analyzing the duration of each segment, as illustrated in Figure 3. Furthermore, the mean, standard deviation, root mean square, and the pseudo-damage ratio [25] of each operating condition relative to the complete dataset are calculated. The results are presented in Table 2. As shown in Table 2, during actual field operations of the combine harvester, the statistical characteristics of signal samples corresponding to different conditions show significant differences, resulting in considerable variations in the pseudo-damage proportions for each condition.

Due to the higher speed and maximum body weight during the full-load field driving condition, although its statistical duration is less than that of the normal harvesting condition, the full-load driving condition contributes the highest damage proportion. Under the normal harvesting condition, the vehicle speed is relatively stable and all working components operate synchronously at high speed, resulting in signals characterized by higher frequency components but smaller amplitudes. The comparison of statistical results between empty-load and full-load field driving indicates that the variation in the weight of the grain tank causes significant differences in the rear axle loading conditions. Additionally, while the adjustment condition during startup and the grain unloading condition have a certain time proportion, their damage contributions are far less compared to other conditions over a complete work cycle.

In summary, the statistical characteristics and damage proportions of various working conditions during the field operation of the combine harvester show significant differences. For agricultural machinery, conducting durability assessments of the entire machine structure using a four-post test rig is an important testing method. Moreover, in the testing process, load spectrum test plans are often developed based on the consistency of damage at target measurement points. In order to improve testing efficiency, it is necessary to optimize the test samples using an appropriate load spectrum acceleration editing method. We retained the conditions with higher damage and reasonably reduced those with smaller damage proportions.

## 3. Theoretical Background

Due to the complexity of operating conditions and the specificity of operational behavior, the load of agricultural machinery equipment mostly exhibits time-varying and non-stationary characteristics, posing challenges for the signal processing of the agricultural machinery load spectrum [26]. Fourier transform is a classical method for the frequency domain analysis of time series. Due to its inherent limitations in time-frequency resolution, it is more suitable for analyzing stationary signals. Wavelet transform uses wavelet functions in the time-scale domain to perform inner product operations with signals. The translation and scale transformation capabilities of the wavelet function allow this method to more accurately describe the local characteristics of signals. It can achieve time subdivision in high-frequency regions and frequency subdivision in low-frequency regions of the signal, making it more suitable for processing non-stationary signals.

**Figure 4:**
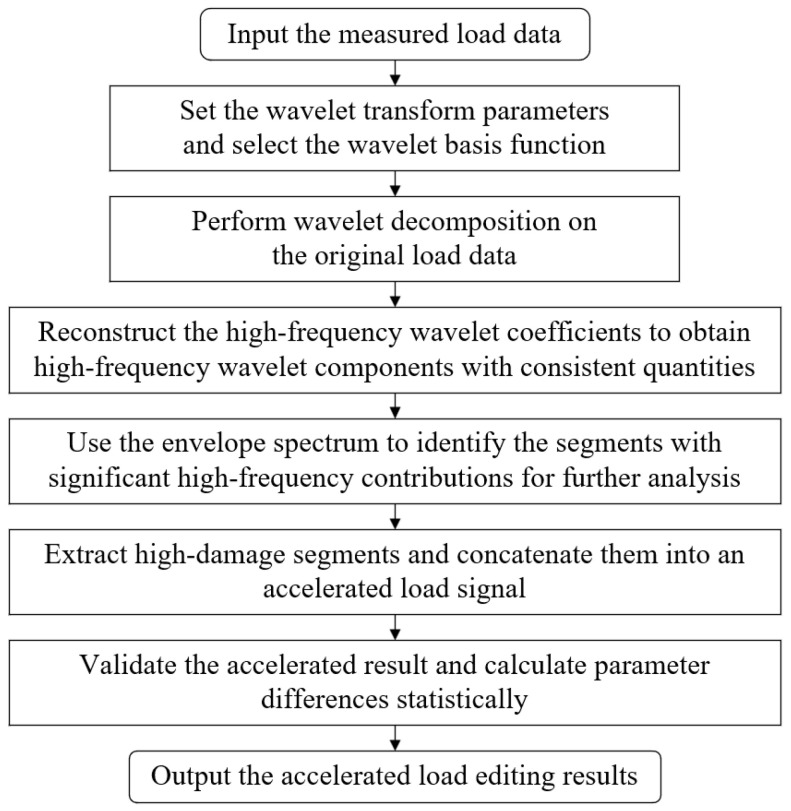
General process of the load spectrum acceleration editing method based on wavelet transform.

The principle of the wavelet transform-based accelerated editing method is to decompose the original time-domain signal using wavelet transform. On this basis, the envelope damage identification method is used to identify and filter out low-damage segments at each level. This ultimately achieves the accelerated editing of the load spectrum in the time domain while preserving its characteristics in the frequency domain [27]. The basic process of this method is shown in Figure 4.

### 3.1. Wavelet Transform and Signal Decomposition

Wavelet transform can use finite-length and decaying wavelet basis functions to convert the original signal into a linear combination of wavelet functions at different scales and shifts. The coefficients of each term together form the wavelet coefficient matrix $W_{\psi }(a,\tau )$. The theoretical calculation formula of continuous wavelet transform is as follows:

(1) $$W_{\psi }(a,\tau )=\frac{1}{\sqrt{a}}\int_{-\infty }^{\infty }x(t)\cdot \psi^{*}(\frac{t-\tau }{a})dt$$

In this formula, *x*(*t*) represents the time-domain signal utilized for computation. $\psi_{a,b}(t)$ is the wavelet basis function. $\psi^{*}$ and ψ are complex conjugates of each other. *a* is the scale parameter, controlling the dilation and compression of the wavelet function, and it is inversely proportional to the frequency. *τ* is the time parameter, controlling the translation of the wavelet function.

Daubechies wavelets are well-known orthogonal wavelet basis functions that have been widely used in the fault diagnosis, noise analysis, and fatigue analysis of non-stationary signals [28]. Therefore, this paper utilizes the Daubechies wavelet function to analyze the stress signal, and with reference to the related research in the automotive field, sets the initial vanishing moment of the Daubechies wavelet function at 12.

### 3.2. Pseudo-Damage Theory

Precise damage assessment and life prediction often require the calibration of fatigue stress cycle curves at targeted measurement points through bench testing, namely the S-N curves, which demand a large number of samples and entail high testing costs. Pseudo-damage theory has been proven to reflect to a certain extent the potential of external excitation loads to damage structural components [29]. By calculating pseudo-damage, it is possible to quantitatively assess the severity of damage between different load segments when the S-N curve for the components is unknown. Therefore, this paper conducts a quantitative assessment of damage to the load signals before and after acceleration editing based on pseudo-damage theory, with the methods described as follows.

Using the rainflow counting method, we can extract information from each load cycle in the actual load measurements. Based on the modified Miner’s criterion, we can accumulate the damage values of all load cycles.

(2) $$D=\sum_{i=1}^{z}\frac{1}{\delta_{i}}=\frac{1}{C}\sum_{i=1}^{z}S_{i}^{k}$$

In the formula, 1/*δ_i_* represents the damage value caused by the *i*th load cycle as measured, *z* is the total number of load cycles, *k* is the inverse slope coefficient of the material’s S-N curve, *S_i_* is the amplitude of the *i*th load cycle, and *C* is a constant variable.

Since there is no strict correspondence between pseudo-damage and material structural parameters, the pseudo-damage *d* can be simplified to the following equation.

(3) $$d=\sum_{i=1}^{z}S_{i}^{k}$$

It is evident that the pseudo-damage *d* is only related to the load cycle amplitude and the inverse slope coefficient *k*, which reflects the fatigue characteristics of the material. For welded structural components or study subjects focused on crack propagation, *k* is set to 3. For typical components with rough surfaces, *k* is set to 5. For components with smooth surfaces, *k* is set to 7 [25]. Since the tested part in this study is a structural component formed by welding multiple steel plates, the material coefficient is set to 3.

We calculate the pseudo-damage for both the original load and the load after acceleration editing, and we then determine the proportion of pseudo-damage *Q* corresponding to different acceleration results. The closer this coefficient is to 1, the higher the consistency of damage between the accelerated signal and the original load.

(4) $$Q=d/d_{0}\times 100\%$$

In the formula, *d*_0_ represents the pseudo-damage corresponding to the original load.

### 3.3. Analysis of Acceleration Editing Results Based on the General Method

**Figure 5:**
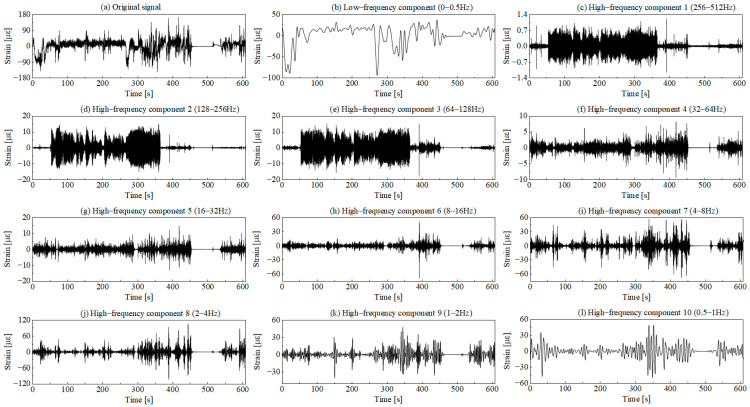
Wavelet decomposition results of the original signal.

The wavelet transform method is employed to decompose the original signal. The decomposition layers are set to 10 based on the spectral distribution characteristics of the original signal. A total of ten high-frequency components and one low-frequency component are obtained, with the decomposition results shown in Figure 5.

**Figure 6:**
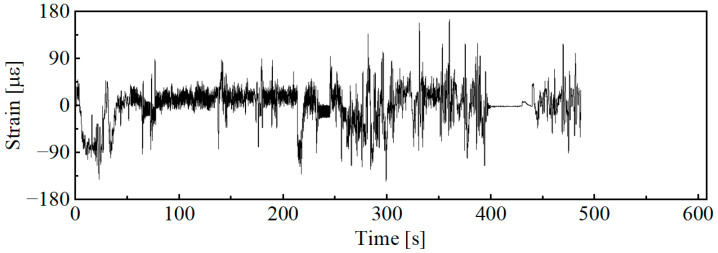
Acceleration editing results for Q ≥ 98%.

The criteria for the output of the acceleration editing results are set to a pseudo-damage ratio *Q* ≥ 98%, with the results of the general method-based acceleration editing shown in Figure 6. It can be seen that the acceleration editing method based on wavelet transformation yields a more streamlined result, reducing the signal length by about 20% compared to the original signal, with a pseudo-damage ratio of 98.41%. However, the acceleration results still retain very small amplitude segments corresponding to certain unloading conditions. This phenomenon indicates that traditional acceleration editing methods based on wavelet transformation are better suited for non-stationary dynamic signals, with further optimization needed in identifying low-damage segments of relatively stable small-amplitude loads.

The main cause of errors in identifying low-damage segments is that the wavelet components after signal decomposition do not align with the frequency domain features of the original signal. When identifying low-damage segments of wavelet components, traditional methods define threshold gradients according to the amplitude range of each component, followed by intersecting these identification results. For stable, small-amplitude signal segments, this approach results in a higher proportion of amplitude in the high-frequency components of the wavelet, potentially leading to identification errors. As shown in Figure 5, the amplitude ratio in Component 1 for the unloading condition is significantly higher than in other components.

To address the above issues, this paper intends to optimize the traditional wavelet-based acceleration editing method by focusing on the selection of characteristic components and threshold division, aiming to improve the applicability of the traditional approach.

## 4. The Improved Wavelet Transform Accelerated Editing Method

### 4.1. Classification of Wavelet Components

**Figure 7:**
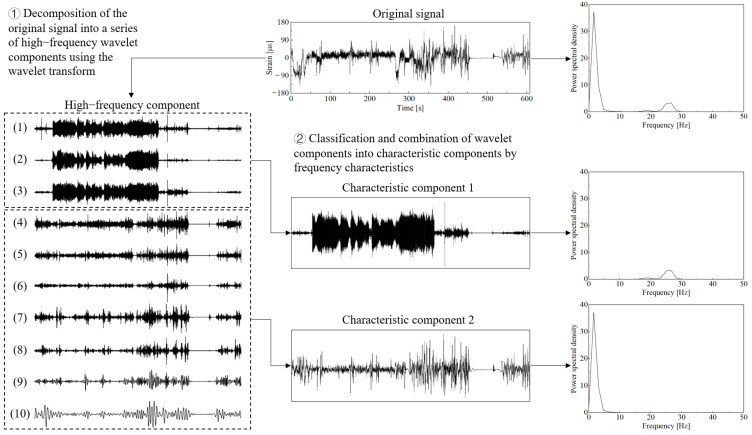
Schematic diagram of the wavelet decomposition and grouping process of the original signal.

The wavelet decomposition process is equivalent to applying multiple continuous band-pass filters to the original signal. The wavelet components obtained through decomposition characterize the signal variations at the target measurement point within specific frequency bands. Since the frequency bands in wavelet decomposition are set layer by layer in a continuous manner, the wavelet components obtained from the decomposition often differ significantly from the frequency-domain characteristic components of the original signal. The time-domain variation curves of wavelet components in Figure 5 reveal two distinct groups of signal characteristics as follows: components 1–3 and components 4–10. Among them, component 1 and component 4, respectively, represent the high-frequency parts of the two groups of characteristic signals. Lower amplitude fluctuations indicate that the component’s damage contribution is minor. If analyzed independently, with the same weight for damage segment identification as the other components, it may introduce errors in damage segment recognition. Therefore, this paper further conducts power spectral density analysis on each wavelet component obtained through decomposition. Based on the operational characteristics of the machine, a series of wavelet components are grouped to more accurately cover the desired characteristic frequency domain, as shown in Figure 7.

According to the frequency domain analysis results shown in Figure 7, the stress signal on the combine harvester chassis can be roughly divided into two parts. Components 1–3 represent the stress variations transmitted to the chassis due to the interaction between the combine harvester’s working parts and crops. This part of the stress has frequency components concentrated in the range of 10–30 Hz. Components 4–10 are mainly caused by road surface irregularities, with the frequency components concentrated in the range of 0–5 Hz. Based on this, the wavelet decomposition results are further divided into characteristic component 1, consisting of components 1–3, and characteristic component 2, consisting of components 4–10. In the subsequent damage segment identification stage, calculations are carried out only for the characteristic components, aiming to avoid damage identification errors caused by wavelet components with insignificant frequency-domain characteristics.

### 4.2. Improved Threshold Selection Method

The primary step in damage segment identification for each characteristic component using the envelope is to define the threshold. The traditional threshold selection method uses the maximum amplitude of each component as the boundary. Let the total number of thresholds involved in the calculation be denoted as *n*, and the positive thresholds for the *i*-th component *D_i_* can be calculated using the following formula. The negative threshold is the opposite of the positive threshold.

(5) $$\mu_{k}=\frac{\max\left(D_{i}\right)}{n}\times k,k=1,2,\cdots ,n$$

**Figure 8:**
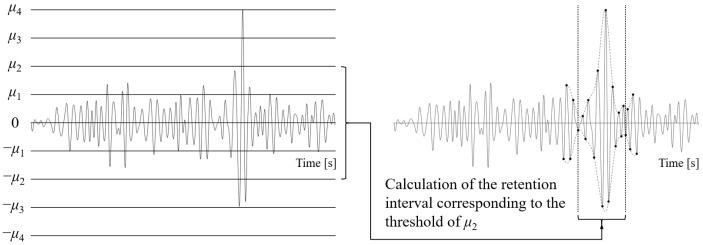
Equidistant threshold selection method and schematic diagram of envelope identification.

At the target threshold level, the portion of the component that exceeds the threshold is identified, and the signal segments to be retained are determined based on the monotonicity of the envelope. As shown in Figure 8, Ts1 and Te1 represent the retained intervals determined by the upper envelope, while Ts2 and Te2 represent the retained intervals determined by the lower envelope. The union of these intervals provides the final retained interval for the current component.

Furthermore, by taking the union of the retained intervals obtained for each component, the signal acceleration editing result at this threshold level is derived. Although the traditional threshold selection method is computationally simple, from a damage perspective, the retained interval results corresponding to the threshold gradient change for each component are not linear. As shown in Figure 5, the maximum amplitude in the sixth wavelet component corresponds to a short-term impact load, and the amplitude variations in the rest of the component differ significantly from the load at the maximum amplitude. If the equidistant threshold selection for components is based on the maximum amplitude, the calculation results corresponding to lower threshold levels show significant differences, while at higher threshold levels, the results will be nearly identical. On this basis, the union of the retained intervals from different components at the same threshold level is taken, leading to errors in identifying low-damage segments in the original signal, which limits the effectiveness of the acceleration editing.

To address the aforementioned problem, this study presents an improved method for threshold selection. By incorporating pseudo-damage theory, it establishes a correlation between the thresholds of each component and the damage ratio of the calculated results in the retention interval. The detailed procedure is as follows:

(1) For the target component, the traditional threshold selection method is used to sequentially calculate the retained signal results corresponding to each threshold level.
(2) Use the rainflow counting method to statistically analyze the load cycle information of the above results. Calculate the pseudo-damage of the retained signal for each threshold level and establish a correspondence between the threshold and the pseudo-damage of the retained interval results.
(3) Following the principle of equidistant increments in pseudo-damage, calculate the new threshold gradient using linear interpolation.

**Figure 9:**
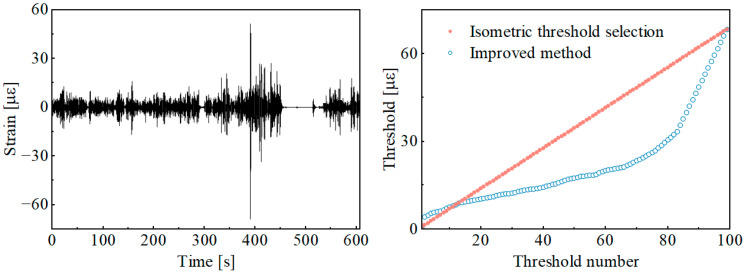
Comparison of results for the threshold selection method.

Figure 9 presented the threshold calculation results derived from different threshold selection methods for wavelet component 6 in Figure 5, with the total number of thresholds set to 100.

As shown in Figure 9, the improved threshold selection method effectively sets thresholds based on the damage gradient from the identification results of damage segments. Compared with the traditional equidistant threshold selection method, the threshold selection method based on the pseudo-damage equidistance principle is better aligned with the logic of damage segment identification during the load spectrum accelerated editing process.

## 5. Results Analysis and Discussion

### 5.1. Time-Domain Characteristic Analysis of Signal Editing Results

**Figure 10:**
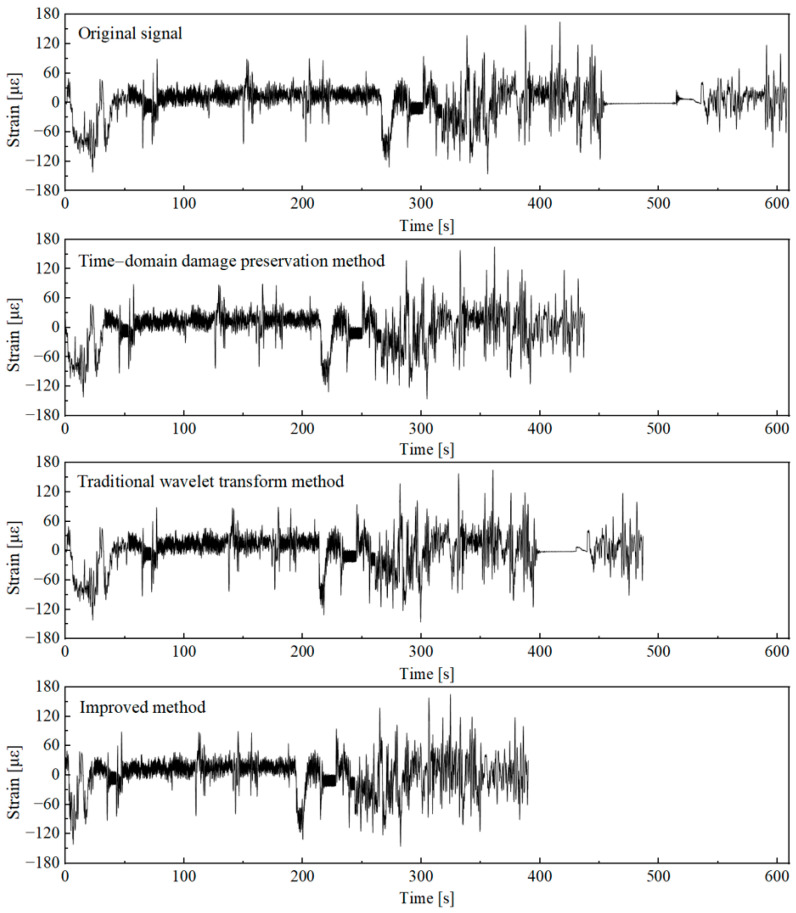
Comparison of time-domain signals of acceleration editing results using different methods.

The signal was analyzed using the improved wavelet transform acceleration editing method, with the wavelet decomposition level set to 10. The high-frequency components were combined based on their frequency-domain characteristics, and the combined feature components were thresholded using the improved threshold selection method. Similarly, the criterion for determining the pseudo-damage ratio of the acceleration editing result was set to *Q* ≥ 98%, obtaining the signal processing results based on the improved wavelet transform acceleration editing method. To fully compare the signal processing effects of different acceleration editing methods, the original signal was further edited using the time-domain damage retention method [10]. This method was implemented using the current mainstream fatigue analysis software, Ncode (2019 version). The window analysis length of the time-domain damage retention method was set to 4 s, based on the signal’s spectral characteristics and the minimum sampling period required to reflect the main frequency components. The signal acceleration editing results were calculated under the condition of a pseudo-damage retention ratio of 98%. The analysis results obtained by different methods are shown in Figure 10, and the pseudo-damage ratio and signal compression ratio compared with the original signal are shown in Table 3.

As shown in Figure 10, it is clear that the accelerated editing results using the traditional wavelet transform method still retain the smooth signal segments that approximate a straight line in the unloading condition. Compared to the traditional wavelet transform method, the improved wavelet transform acceleration editing method can more accurately filter out the stationary low-amplitude signal segments corresponding to the unloading conditions. Under a pseudo-damage ratio of 98.18%, the time-domain length of the accelerated signal is only 64.16% of the original signal. The time-domain damage retention method also shows excellent performance in identifying low-damage signal segments under unloading conditions. However, the fixed analysis window in the method limits its acceleration effectiveness when processing non-stationary signal segments. Under a pseudo-damage ratio of 98.07%, the time-domain length of the accelerated signal is 71.94% of the original signal. Under the same pseudo-damage ratio level, the improved wavelet transform acceleration editing method enhanced the acceleration effect in time-domain signal reduction by 15.92% and 7.76% compared to the traditional method and the time-domain damage retention method, respectively. This means that if a 100 h bench test is to be conducted, the improved method can save up to approximately 16 h of testing time, which has significant practical value for reducing test costs and improving testing efficiency.

A further analysis was performed on the statistical characteristic parameters of acceleration results under different acceleration editing methods, such as root mean square (RMS) values and kurtosis coefficients, to quantitatively assess the time-domain characteristic differences between the accelerated and original signals. The root mean square (RMS) value can partially reflect the average energy contained in the signal. The kurtosis coefficient, also known as the peakedness coefficient, reflects the degree of concentration of the data. A higher kurtosis indicates that the probability density distribution curve of the signal is sharper at the mean value. By comparing the consistency of the statistical characteristics of signals before and after acceleration editing, the effectiveness of each method was evaluated to ensure consistency in terms of average energy and distribution characteristics between the accelerated signal and the original signal. The results are shown in Table 4.

As the time-domain signals undergo varying degrees of editing and reduction, statistical parameters between the accelerated signal and the original signal show deviations. The magnitude of these deviations is related to the logic of low-damage segment reduction and the final compression ratio of the accelerated signal in each acceleration editing method. As shown in Table 4, the accelerated signals derived from different methods exhibit higher RMS values and lower kurtosis coefficients compared to the original signal. The reason lies in the fact that the low-amplitude stress cycles, which contribute less to damage in the analyzed signal, are primarily concentrated under unloading conditions and field adjustment conditions. Under unloading conditions, the stress values are smaller, and as these signal segments are reduced, the RMS value of the accelerated signal increases. The reduction in the number of samples concentrated around the mean value leads to a decrease in the kurtosis coefficient.

Since the purpose of load spectrum acceleration editing is to filter out low-damage contribution segments in the original signal, the consistency of statistical parameters between the accelerated signal and the original signal does not necessarily reflect the quality of the acceleration results. Compared with the time-domain damage retention method and the improved wavelet transform method, the traditional wavelet transform method has errors in identifying low-damage segments under unloading conditions. Although the statistical parameters are relatively consistent, the acceleration effect is poor. Compared to the time-domain damage retention method, the improved wavelet transform method achieves more compact acceleration editing results with smaller deviations in statistical parameters, confirming the accuracy and effectiveness of the proposed improved method in identifying low-damage segments.

### 5.2. Power Spectral Density Analysis

**Figure 11:**
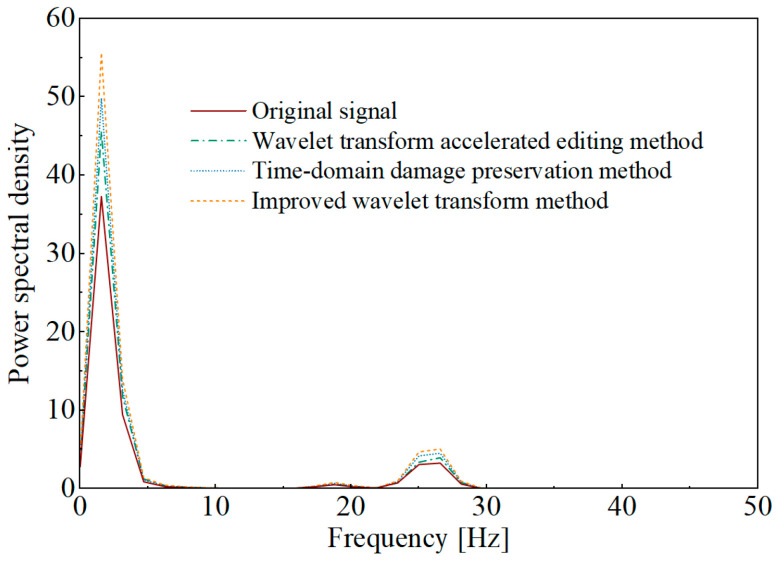
Comparison of the PSD of the acceleration editing results.

Power spectral density (PSD) reflects the energy distribution of a signal across frequencies and represents the energy per unit time per unit frequency. Under the 98% pseudo-damage ratio criterion, the accelerated signals obtained by the three methods and the original signal were subjected to power spectral density analysis. The comparison results are shown in Figure 11.

Figure 11 indicates that the PSD analysis results of the accelerated signals obtained using the three methods align with the overall trend of the PSD of the original signal. The PSD analysis results of the accelerated signals show slightly higher amplitudes at the aforementioned main frequencies compared to the original signal, indicating that the signal components corresponding to these frequency ranges have a higher damage contribution ratio and are more significantly retained during the acceleration editing process. This leads to an increase in the average power within each unit frequency bandwidth, which in turn results in higher calculated power spectral density values.

### 5.3. Influence of the Wavelet Function Vanishing Moments on the Effect of Accelerated Editing

**Figure 12:**
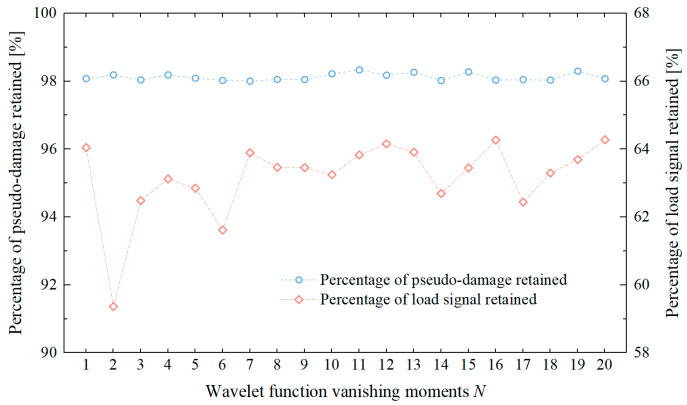
Comparison of acceleration results under different wavelet function vanishing moment conditions.

The vanishing moment of a wavelet function reflects the smoothness and concentration of the wavelet function in the time and frequency domains. In the previous sections, the Daubechies wavelet function with a vanishing moment of 12 (db12) was used to conduct a comparative analysis of wavelet-based acceleration editing methods, verifying the effectiveness of the improved wavelet transform acceleration editing method. Currently, there is no literature discussing the effect of changes in the vanishing moment of the wavelet function on the signal acceleration editing results in the field of agricultural machinery. The basis for setting the vanishing moment remains insufficient. Therefore, this study further analyzed the load spectrum using the improved wavelet transform acceleration editing method under conditions where the vanishing moment *N* = 1–20. The pseudo-damage ratio determination criterion was set as *Q* ≥ 98%, and the calculation results of the pseudo-damage ratio and signal compression ratio of the accelerated signal as a function of the wavelet function’s vanishing moment are shown in Figure 12.

As shown in Figure 12, under the same pseudo-damage ratio criterion, changes in the wavelet function’s vanishing moment significantly affect the signal compression ratio of the accelerated signal. The fluctuation range of the time-domain signal length is approximately 5%. Compared to the initially set db12 wavelet function, the db2 wavelet can achieve accelerated editing results, with a signal retention ratio of 59.36%, while essentially maintaining the same damage retention ratio (*Q* = 98.19), improving the acceleration effect by an additional 4.8%. During indoor accelerated durability testing, the edited load spectrum often needs to undergo multiple cyclic loadings. Therefore, under the condition of consistent damage, selecting a load spectrum with a more compact time-domain length has a significant impact on improving test efficiency.

## 6. Conclusions

This paper proposes a load spectrum acceleration editing method based on wavelet transform. By classifying and merging wavelet components according to their frequency components, the method participates in accelerated editing and optimizes the threshold selection approach to enhance the acceleration editing effect for the stress signal of the combine harvester chassis. On this basis, the influence of wavelet function parameter selection on the acceleration editing results is discussed. This study can provide beneficial experiential references for load spectrum development in the accelerated durability testing of agricultural machinery. The specific research conclusions are as follows:

(1) In traditional wavelet transform acceleration editing methods, since the signal frequency components and statistical characteristics represented by different wavelet components vary significantly, identifying damage segments with equal weight for all wavelet components without distinction will have a negative impact on the acceleration results, especially when dealing with stationary low-amplitude signals. Classifying and combining wavelet components based on frequency characteristics and reasonably dividing thresholds according to the principle of pseudo-damage equidistance is an effective improvement approach.
(2) Compared to the time-domain damage retention method and traditional wavelet transform acceleration editing methods, the proposed improvement in this paper achieves more streamlined acceleration results under the same pseudo-damage ratio condition (*Q* ≥ 98). The time-domain signal length is reduced by 7.76% and 15.92%, respectively. Moreover, the accelerated signal maintains consistency with the original signal in terms of statistical parameters (RMS value and kurtosis coefficient) and power spectral density.
(3) Under consistent pseudo-damage ratio determination conditions, the vanishing moments of wavelet functions are also important parameters influencing the acceleration editing results. For the improved wavelet transform acceleration editing method, compared to the initially set db12 wavelet function, the db2 wavelet can further reduce the time-domain signal length by 4.8%. Therefore, it is recommended to supplement the variable analysis of wavelet function vanishing moments when applying wavelet transform acceleration editing methods to obtain a more optimized load spectrum.

## Figures and Tables

**Table 1:** Sensor and related hardware parameters.

Name	Model	Technical Specifications
Strain Gauge	BE-350-3BB(11)-Q30	Resistance value 350 Ω
		Sensitivity factor 2.10 ± 1%
Data Acquisition Device	SoMat eDAQ	32-channel EBRG
		Sampling rate ≤ 100 kHz

**Table 2:** Statistical parameters and damage proportions of each operating condition.

Operating Condition	Mean	Standard Deviation	Root Mean Square	Percentage of Pseudo-Damage
1—Startup adjustment	46.99	42.09	63.08	2.5%
2—Normal harvesting	12.77	16.41	20.79	30.9%
3—Field reversing	21.03	38.20	43.60	5.5%
4—Field driving with a full tank	0.38	43.25	43.25	52.1%
5—Grain unloading	6.97	22.61	23.66	0.7%
6—Field driving with an empty tank	−1.20	7.22	7.32	8.2%

**Table 3:** Pseudo-damage ratio and signal compression ratio for the acceleration results of different methods.

Method	Pseudo-Damage Ratio	Signal Compression Ratio
Time-domain damage retention method	98.07%	71.94%
Traditional wavelet transform method	98.42%	80.08%
Improved wavelet transform method	98.18%	64.16%

**Table 4:** Statistical parameter comparison of acceleration results obtained by different methods.

Method	RMS/με (Deviation/%)	Kurtosis Coefficient (Deviation/%)
Original signal	32.94	5.41
Time-domain damage retention method	37.12 (12.67%)	4.32 (20.19%)
Traditional wavelet transform method	35.41 (7.49%)	4.70 (13.19%)
Improved wavelet transform method	36.64 (11.24%)	4.56 (15.69%)

## Data Availability

Data will be made available upon request.
